# Updating the list of known opioids through identification and characterization of the new opioid derivative 3,4-dichloro-N-(2-(diethylamino)cyclohexyl)-N-methylbenzamide (U-49900)

**DOI:** 10.1038/s41598-017-06778-9

**Published:** 2017-07-24

**Authors:** D. Fabregat-Safont, X. Carbón, M. Ventura, I. Fornís, E. Guillamón, J. V. Sancho, F. Hernández, M. Ibáñez

**Affiliations:** 1 Research Institute for Pesticides and Water, University Jaume I, Avda. Sos Baynat s/n, 12071 Castellón, Spain; 2 Energy Control (Asociación Bienestar y Desarrollo), c/Independencia 384, 08041 Barcelona, Spain; 3 Departament de Química Física I Analítica, University Jaume I, Avda. Sos Baynat s/n, 12071 Castellón, Spain

## Abstract

New psychoactive substances have been rapidly growing in popularity in the drug market as non-illegal drugs. In the last few years, an increment has been reported on the use of synthetic alternatives to heroin, the synthetic opioids. Based on the information provided by the European Monitoring Centre for Drug and Drug Addiction, these synthetic opioids have been related to overdoses and deaths in Europe and North America. One of these opioids is the U-47700. A few months ago, U-47700 was scheduled in the U.S. and other countries, and other opioid derivatives have been appearing in order to replace it. One of these compounds is U-49900, an analog of U-47700. A white powder sample was obtained from an anonymous user in Spain. After an accurate characterization by gas chromatography-mass spectrometry, ultra-high performance liquid chromatography-high resolution mass spectrometry, nuclear magnetic resonance and single-crystal X-ray diffraction; and complemented by Fourier-transformed infrared spectroscopy, ultraviolet and circular dichroism spectrophotometry, the drug sample was unequivocally identified as U-49900. The information provided will be useful for the Early Warning System and forensic laboratories for future identifications of the U-49900, as well as in tentative identifications of other related opioids.

## Introduction

**Figure 1:**
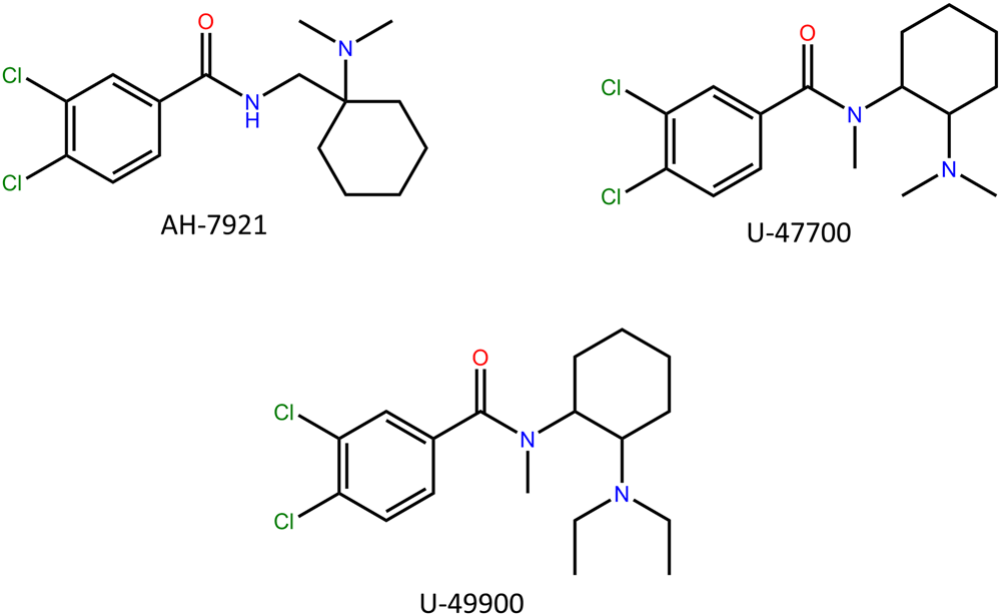
Chemical structure for AH-7921, U-47700 and expected structure for U-49900.

In recent years, new psychoactive substances (NPS) have rapidly emerged in the drug market as a “legal” alternative to controlled drugs^1, 2^. The term “NPS” includes different compound families, such as synthetic cannabinoids, cathinones and opioids. In the last decade, an increase in the use of synthetic opioids as substance of abuse has been reported^3^, with the potential to pose serious risks to public health and safety. According to the EMCDDA *European Drug Report 2016*, synthetic opioids have been involved in overdose drug related deaths in some parts of Europe and North America, these compounds having been found in 82% of fatal overdoses^4^. For years, the diversity of available synthetic opioids in the grey market has been limited. Fentanyl analogues are limited by their high potency, making them unwieldy and dangerous to handle by users without proper safety procedures; thus, opioids such as MT-45 have been demonstrated to cause dangerous side effects^5^. Around 2012, a new class of synthetic opioids emerged. AH-7921 (Fig. 1), an opioid around 0.8 times as potent as morphine, developed at Allen & Hanburys Ltd in 1975^6^, resurfaced on internet stores sold under the guise of being a research chemical “not for human consumption”. Effects described by users were similar to those of classical opioids: euphoria, sedation, pinned pupils, etc.^7^, and it saw relatively little popularity until its ban in 2013. Shortly after, an analogue of AH-7921 appeared on the market, U-47700 (Fig. 1), developed by Upjohn in 1978^8^. In the Upjohn patent, U-47700 is the compound most selective for the mu opioid receptor among all studied. It’s theorized to have a potency of around 7.5 times that of morphine, and binds to the mu opioid receptor (MOR) with a Ki (±SEM) of 0.91 nM (±0.11), to kappa opioid receptor (KOR) at 110 nM (±11), and poorly so to the delta opioid receptor (DOR) at 480 nM (±110). In comparison, morphine has Ki (±SEM) values at the MOR, KOR, and DOR of 0.213 (±0.019), 27.9 (±2.7), and 111 (±14) nM, respectively.

U-47700’s presents a certain set of characteristics that make it particularly attractive to users, that until its appearance no other single substance had had. It is described by users as being more euphoric than fentanyl analogues, more potent than AH-7921, cheap, and widely available (at the time of writing this article)^9^. It is no surprising that it saw a surge in popularity, and has been the cause of at least 46 confirmed fatalities, as well as 88 reports from forensic laboratories of U-47700 submissions in the U.S. alone^10^, as well as several fatalities in the rest of the globe^11–16^. With a common dose being six to eight milligrams, the cost per dose is no more than 0.32 USD^17^. As a response to its growing popularity, the DEA placed U-47700 in emergency scheduling along with furanyl-fentanyl in November 11th, 2016^10^.

The NPS market is quick to adapt however, and shortly before U-47700 was scheduled, U-51754, another synthetic opioid from the same Upjohn patent surfaced on the market. This substance is not as selective for KOR, and users describe it as being more dysphoric and dissociating than U-47700^18^. The most recent compound to surface, and a potential replacement for U-47700, is U-49900. Although both substance names bear a striking similarity, U-49900 does not appear on the same Upjohn patent as U-47700 or U-51754, and is in fact a completely novel substance that has not been described in literature until now. In this work, a sample (labelled as U-49900) was obtained by Energy Control from an anonymous Spanish user. After an exhaustive analysis by gas chromatography coupled to single-quadrupole mass spectrometry (GC-MS), ultra-high performance liquid chromatography coupled to high-resolution mass spectrometry (UHPLC-HRMS), nuclear magnetic resonance (NMR) and single-crystal X-ray diffraction, the compound was unequivocally characterized and thus, identified. Additionally, Fourier-transformed infrared (FTIR) spectroscopy, ultraviolet (UV) analysis, circular dichroism (CD) and melting point range determination were performed in order to obtain a fully-characterization of the compound. This methodology has proved to be powerful for the identification of NPS, even for unknown substances^19^. The compound was confirmed to be the diethyl analogue of U-47700. In this work, we present the detailed analytical characterization of 3,4-dichloro-N-(2-(diethylamino)cyclohexyl)-N-methylbenzamide, henceforth referred to as U-49900 (Fig. 1). Available information about its effects, price, availability, etc. has also been included.

## Results

### Analytical characterization of U-49900

#### Gas chromatography-quadrupole mass spectrometry

**Figure 2:**
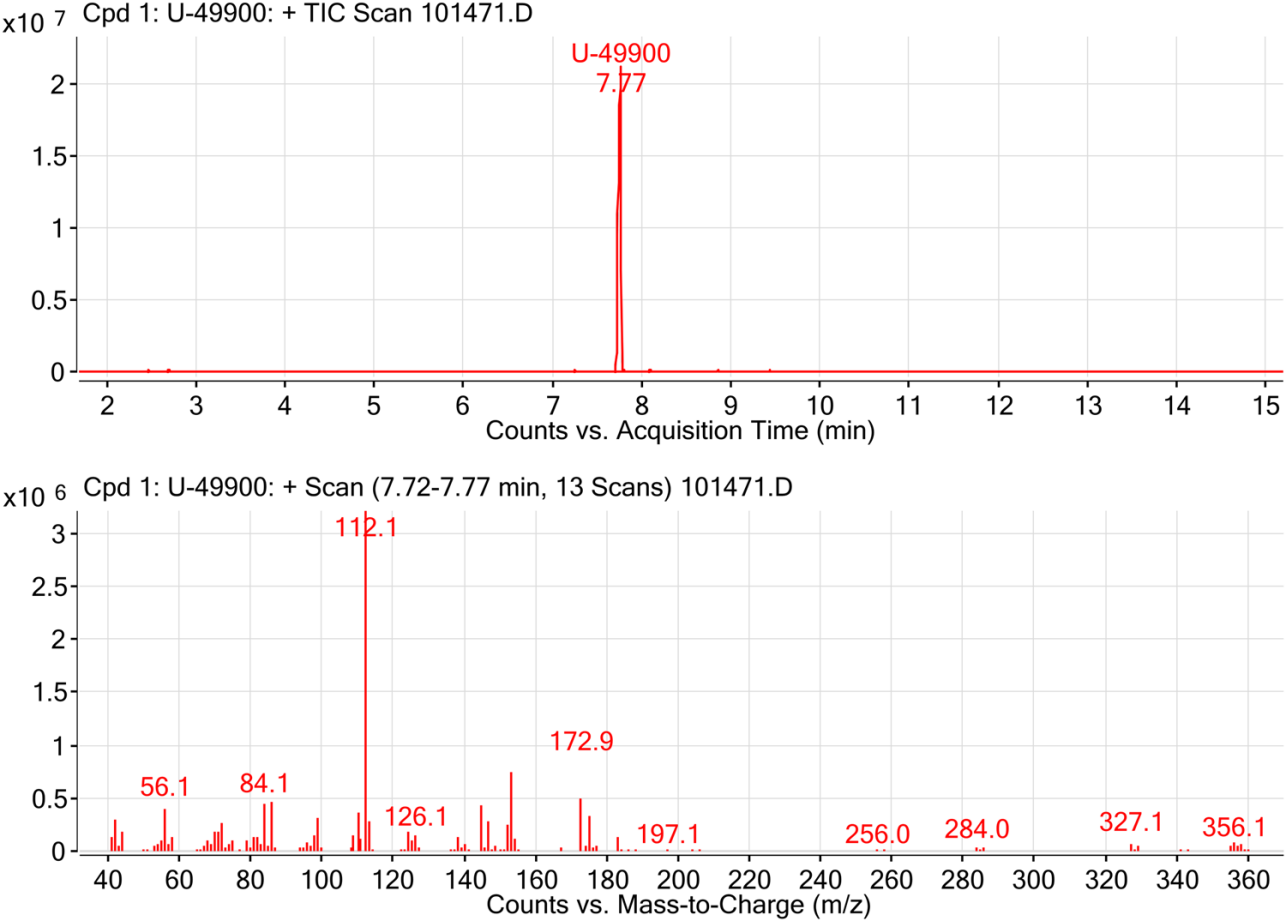
GC-EI-MS analysis of the sample containing U-49900. Top: TIC of the sample analysis. Bottom: EI spectrum for the chromatographic peak at 7.77 min.

Firstly, the sample was analysed by GC-MS. A unique peak at 7.77 min was observed in the Total Ion Chromatogram (TIC), demonstrating the high purity of the sample (Fig. 2, top). The EI spectrum of this peak showed a high fragmentation, being the base peak the ion at *m/z* 112 (Fig. 2, bottom). This peak did not show the expected isotopic pattern produced by the putative presence of two chlorine atoms in the molecule. Additional fragment ions with an important intensity were observed at *m/z* 56, 84, 126, 144, 154 and 173. In this case, the ion at 173 presented the characteristic isotopic pattern corresponding to the presence of two chlorine atoms. Ions above *m/z* 200 were also observed, but with a lower intensity. Ions at *m/z* 284, 327 and 356 also presented the two-chlorine pattern. The molecular ion expected at *m/z* 356 was observed, but with a very low intensity, due to the high fragmentation obtained by EI. Despite all the information provided, this technique did not allow the unequivocal characterization of the molecule and thus, additional techniques were used for the confirmation of the compound.

#### Liquid chromatography-high resolution mass spectrometry

**Figure 3:**
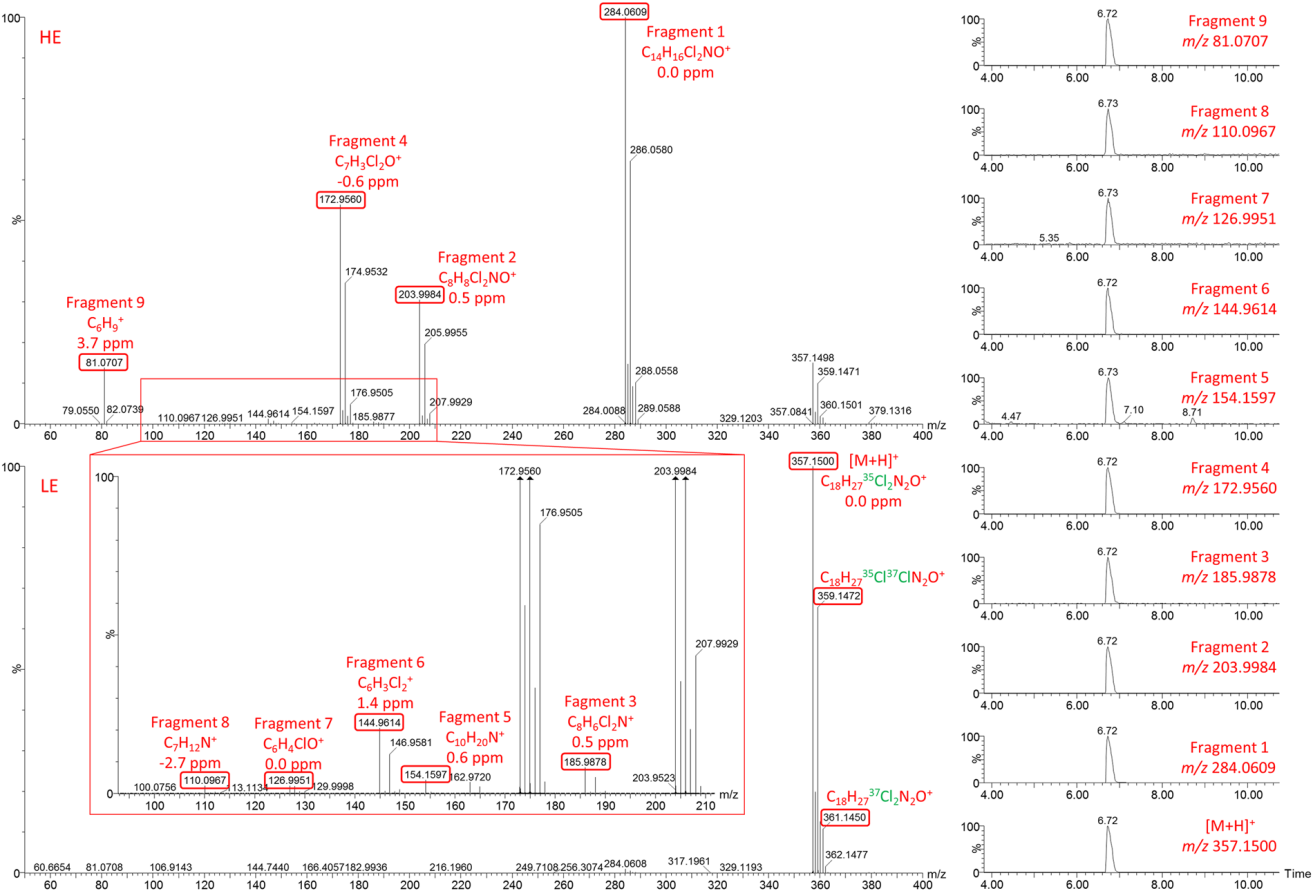
UHPLC-HRMS MSE spectra of the U-49900. Right: Extracted ion chromatograms (EIC) (0.02 Da mass window) for protonated molecule in LE function, and fragment ions in HE function. Left: LE (bottom) and HE (top) spectra of U-49900.

UHPLC-HRMS analysis was performed in order to tentatively identify the compound based on its fragmentation. The soft ionization interface used allows the acquisition of the protonated molecule in the LE spectrum. In Fig. 3 (left) the LE spectrum of the chromatographic peak at 6.72 min is showed. A unique peak at *m/z* 357.1500 was observed, corresponding the protonated molecule ([M + H]^+^) of the compound. The accurate-mass acquisition allowed the determination of the elemental composition of this *m/z* peak, being calculated as C_18_H_27_Cl_2_N_2_O^+^ with a mass error of 0.0 ppm. Other possible elemental compositions were neglected due to the high mass error obtained. The determined elemental composition fitted with the theoretical elemental composition of U-49900. The isotopic pattern of the [M + H]^+^ showed an important peak at *m/z* 359, corresponding to the presence of two chlorine atoms in the molecule. The ratio between both peaks (65% approximately) was the expected for the ratio ^35^Cl_2_/^35^Cl^37^Cl. Similarly, the ratio between [M + H]^+^ and the peak at *m/z* 361 corresponding to the ratio between ^35^Cl_2_/^37^Cl_2_ was also observed. No significant fragment ions were observed in the LE function.

The HE spectrum showed four important collision-induced fragment (CID) ions at *m/z* 284.0609, 203.9984, 172.9560 and 81.0707 (Fig. 3, left). In order to evaluate if this fragment ions came from the protonated molecule, extracted ion chromatograms (EIC) were generated (Fig. 3, right). Ions at *m/z* 284, 204 and 173 showed the isotopic pattern corresponding to the presence of two chlorine atoms. Based on the expected structure of U-49900, these three ions would correspond to fragments which contain the dichlorophenyl group. This assumption was confirmed once their elemental composition was determined. Fragment at *m/z* 284 (C_14_H_16_Cl_2_NO^+^, 0.0 ppm) corresponds to the diethylamino loss; fragment at *m/z* 204 (C_8_H_8_Cl_2_NO^+^, 0.5 ppm) corresponds to the cyclohexyl loss from fragment at *m/z* 284; and fragment at *m/z* 173 (C_7_H_3_Cl_2_O^+^, −0.6 ppm) would be the methylamine loss of fragment 204. The other important fragment, at *m/z* 81 (C_6_H_9_^+^, 3.7 ppm), corresponds to the cyclohexyl ring, whose formation was already observed in the fragment at *m/z* 204 (a cyclohexyl ring loss from fragment *m/z* 284). All these fragments have been previously described in literature for the U-47700, and it was expected that some of them could also be observed for the U-49900^12^.

An accurate examination of the HE allowed the detection of five additional fragment ions, not previously observed due to their low intensity. The ions at *m/z* 185.9878 and 144.9614 also presented the isotopic pattern corresponding to the presence of two chlorine atoms. Fragment at *m/z* 186 (C_8_H_6_Cl_2_N^+^, 0.5 ppm) corresponds to the water loss from fragment at *m/z* 204. Thus, fragment at *m/z* 204 would present two different sequential fragmentations: a water loss (fragment at *m/z* 186) and a methylamine loss (*m/z* 173). The remaining dichlorinated fragment at *m/z* 145 (C_6_H_3_Cl_2_^+^, 1.4 ppm) corresponds to the dichlorophenyl group. This fragment could be produced from fragment *m/z* 173 by a carbon monoxide loss, or from fragment *m/z* 186 by an azirine loss. Both fragments (at *m/z* 186 and 145) have also been described for the U-47700^12^.

Three minor fragments were observed at *m/z* 154.1597, 126.9951 and 110.0967. Fragments at *m/z* 154 (C_10_H_20_N^+^, 0.6 ppm) and *m/z* 110 (C_7_H_12_N^+^, −2.7 ppm) correspond to the diethylcyclohexylamine group (which would come from [M + H]^+^) and the methylcyclohexylamine group (which would come from fragment at *m/z* 284 by C-N amide bond fragmentation), respectively. Additionally, both fragments would be the precursors of fragment at *m/z* 81 (the cyclohexyl group), previously described. Regarding fragment at *m/z* 127 (C_6_H_4_ClO^+^, 0.0 ppm), this fragment would correspond to a 2-chlorophenol group, but this structure is not present in the supposed structure of U-49900. This fragment seems to be produced by an adduct formation with a neutral molecule present in the collision cell. The formation of fragment-adducts with small molecules in the collision cell has been recently described in literature^20^. According to literature, water adducts are the most commonly observed, due to water traces coming from the same instrument or CID gas impurities^20^. Based on this study, the fragment at *m/z* 127 would come from the dichlorophenyl fragment (*m/z* 145), experiencing a neutral HCl loss followed by a water addition, and subsequent formation of the 2-chlorophenol fragment. In this case, no information related with the U-47700 fragmentation was found in literature for fragments at *m/z* 154 (on the basis of the structure, this ion could not be present in U-47700), 127 and 110.

Finally, MS/MS experiments were performed in order to increase the confidence of the CID fragments acquired (spectra can be found in Supplementary Information, SI.1).

**Figure 4:**
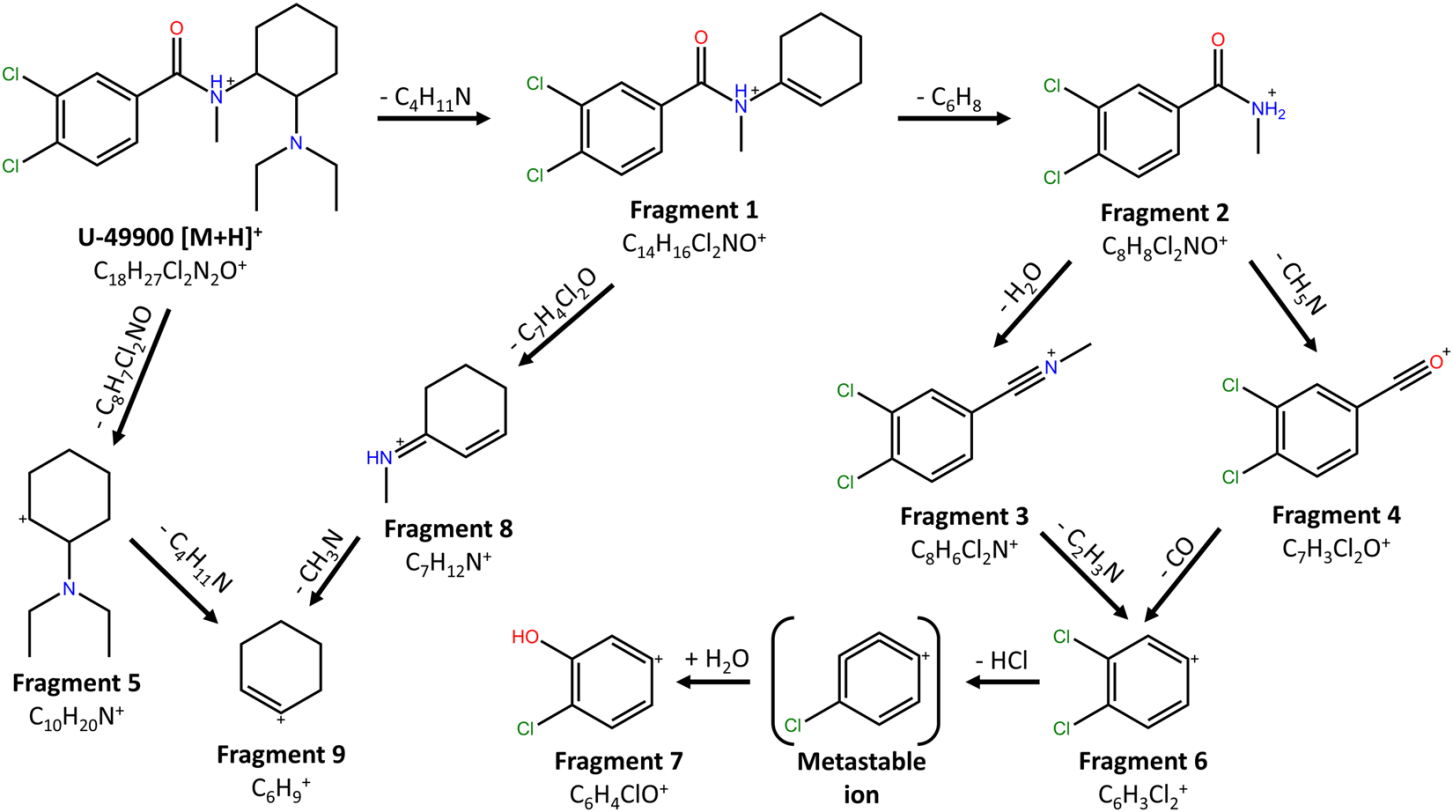
Proposed CID fragmentation pathway for the U-49900.

Once the compound was tentatively identified based on its accurate-mass fragmentation, a plausible fragmentation pathway was proposed (Fig. 4). Nevertheless, a tentative identification was not enough to characterize the compound, thus additional spectroscopic techniques were applied for the identification and subsequent complete characterization of the U-49900.

#### Nuclear magnetic resonance

**Figure 5:**
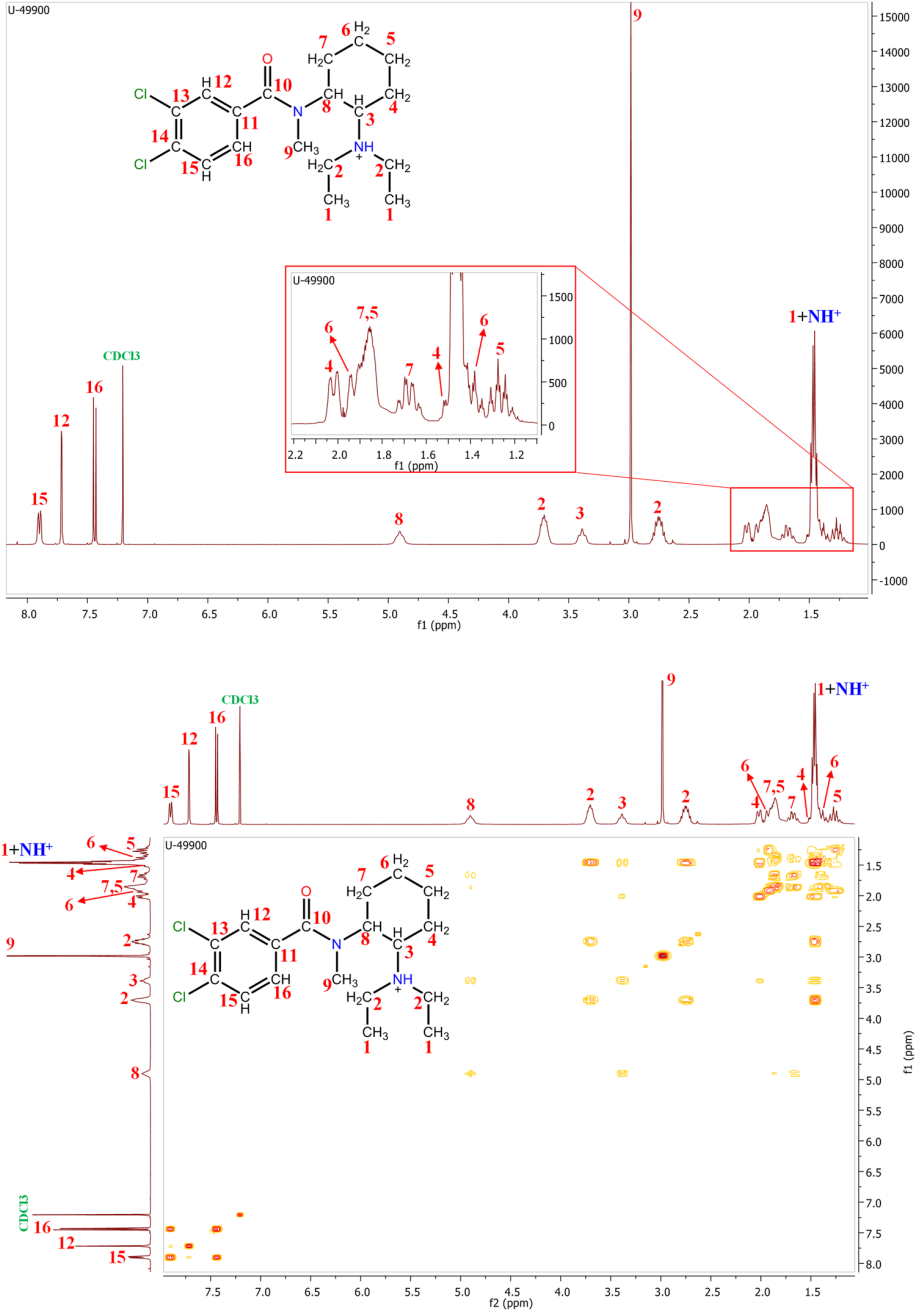
Top: ^1^H-NMR spectrum for U-49900 with signal assignation based on compound structure. Bottom: COSY spectrum of U-49900.

Different NMR experiments were performed in order to identify the compound. The first step was to perform ^1^H-NMR (Fig. 5, top) and COSY (Fig. 5, bottom) experiments (400 MHz, CDCl_3_). COSY are bidimensional NMR experiments (2D-NMR) which shows through-bond correlations between the hydrogens of the molecule. As the compound was tentatively identified by UHPLC-HRMS, most of the NMR signals were easily identified. For ^1^H-NMR, signals between δ = 7.5 and 8.0 ppm corresponded to the aromatic hydrogens, identifying each signal based on its multiplicity and chemical shift. Doublets corresponded to the hydrogens of carbon marked as 15 in Fig. 5 (C15) and C16 hydrogens. The C15 would correspond to the doublet with the highest chemical shift (δ = 7.89 ppm) due to the proximity of the chlorine atom; the C16 would correspond to the other doublet (δ = 7.45 ppm). The third signal, a singlet, corresponded to C12 hydrogen (δ = 7.72 ppm). COSY spectrum showed a correlation between C15 and C16 and no correlation with C12, as expected.

The signal at δ = 4.90 ppm would correspond to the C8 hydrogen based on the multiplicity and chemical shift. Similarly, the signal at δ = 3.39 ppm might correspond to the hydrogen in C3. A correlation between C3 and C8 hydrogens was observed in COSY. The most intense signal in ^1^H-NMR was the singlet at δ = 2.98 ppm, produced by the methyl group named as C9 in Fig. 5, without correlations with other hydrogens.

The CH_3_ of both ethyl groups bonded to the cyclohexylamine were expected to be found as a triplet around δ = 1.5 ppm, with an integration corresponding to 6 hydrogens. Even so, the signal at δ = 1.46 ppm was a quadruplet with an integration of 7 hydrogens. Signals corresponding to protonated amines are expected to be between 0.5 and 5 ppm, and it is possible that the observed quadruplet was produced by a triplet (corresponding to CH_3_) and a singlet (corresponding to NH^+^). In order to prove that, an additional ^1^H-NMR experiment was performed, using deuterated water as solvent (400 MHz, D_2_O). The use of deuterated water as solvent promotes protonatable groups to exchange deuterium from the solvent. These signals from ^1^H-NMR spectrum are therefore removed as deuterium does not produce resonance in ^1^H-NMR. The resulting signal was a triplet whose integration corresponded to 6 hydrogens, therefore confirming our hypothesis. ^1^H-NMR spectra acquired using CDCl_3_ and D_2_O comparison can be found in Supplementary Information SI.2. So, the signal at δ = 1.46 ppm was assigned to the hydrogens of C1 and the proton of the amine (Fig. 5).

Signals at δ = 2.75 and 3.70 ppm would correspond to the CH_2_ of the ethyl groups (C2), but producing different chemical shifts. This would indicate that the ethyl groups presented a different electronic surrounding. The protonation of the amine could produce an intramolecular hydrogen bond between this proton and the oxygen atom or the nitrogen atom of the amide group. The formation of this bond would hamper the free rotation of the N-cyclohexyl bond, producing the diastereotopicity of the C2 hydrogens. Once the signals produced by C2 hydrogens were identified, the correlation with C1 hydrogens was confirmed after performing an accurate study of the COSY spectrum. Finally, low-intense signals between δ = 0 and 2 ppm corresponded to CH_2_ cyclohexyl hydrogens (C4-C7), as is explained in the ^13^C-NMR and HSQC study.

**Figure 6:**
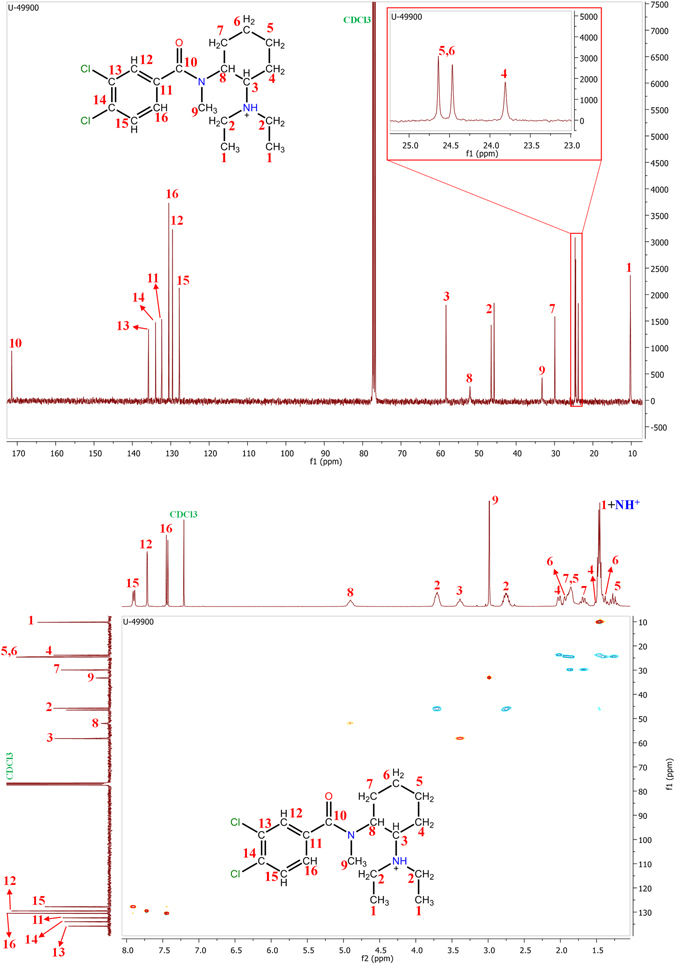
Top: ^13^C-NMR spectrum for U-49900. Bottom: HSQC spectrum of U-49900.

The second step was to perform ^13^C-NMR (Fig. 6, top) and HSQC (Fig. 6, bottom) experiments (400 MHz, CDCl_3_). HSQC is a 2D-NMR experiment which establishes the through-bond correlation between the carbon atoms and their bonded hydrogens. HSQC spectra work with a colour code: CH_3_ and CH groups are represented in one colour, while CH_2_ is represented in another one. In this case, in the HSQC spectrum of U-49900 CH_3_/CH are represented in red-yellow spots, while CH_2_ is represented in blue spots (Fig. 6, bottom). Most ^13^C-NMR signals were assigned based on the assignation previously performed in ^1^H-NMR and looking for the correlation between carbon and hydrogen signals in HSQC. Quaternary carbon atoms, which have no hydrogens and thus no signal in ^1^H-NMR, were identified based on the chemical shift. Signal at δ = 171.46 ppm corresponded to the carbon atom of the amide group (C10). The three signals in the aromatic region (between δ = 100 and 140 ppm) which did not have correlation with ^1^H-NMR signals corresponded to the functionalized carbon atoms of the benzene ring. Signals at δ = 133.97 and 135.80 ppm were assigned to the carbons bonded to chlorine atoms (C13 and C14), while signal at δ = 132.37 ppm corresponded to C11.

An accurate analysis of the HSQC spectrum confirmed the previously explained supposition: the hydrogens of C2 are diastereotopic. ^13^C-NMR spectrum showed a slight change in the chemical shift of both C2 carbons, probably due to the proximity of one of them to the nitrogen atom of the amide group. HSQC also showed the correlation between ^13^C-NMR signals corresponding to C2 carbon, and the two observed signals in ^1^H-NMR related previously to C2 hydrogens, reinforcing the supposition of the amine protonation. Finally, signals corresponding to CH_2_ of the cyclohexyl (signals between δ = 0 and 2 ppm in ^1^H-NMR) presented diastereotopicity. This was observed in HSQC spectrum as a double ^1^H-NMR signal corresponding to a single ^13^C-NMR signal. In other words, there were two different hydrogens in each carbon atom of the cyclohexane. This double signal was easy to justify, as the hydrogens of a CH_2_ in a cyclohexane can present two different orientations: axial and equatorial. The different orientation of the hydrogens produces diastereotopicity in hydrogens theoretically equivalents.

**Figure 7:**
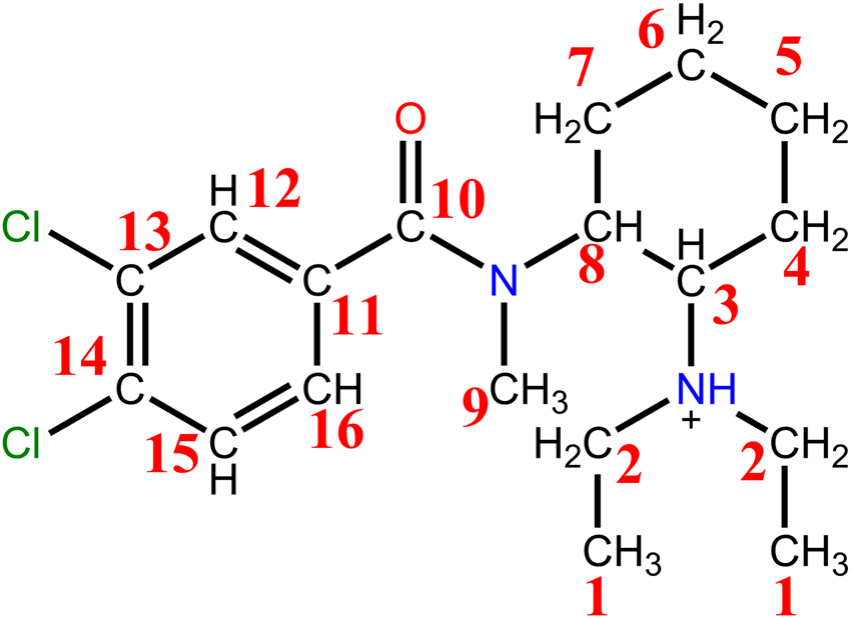
^1^H and ^13^C-NMR signal assignment.

The combination of COSY and HSQC spectra allowed a complete NMR characterization of the compound, increasing the confidence on the tentative identification of the structure of U-49900. Table 1 and Fig. 7 show the assignation of ^1^H and ^13^C-NMR signals once stablished the correlation between atoms and signals.

**Table 1:** ^1^H and ^13^C-NMR signal assignment.

^1^H-NMR signal assignment			^13^C-NMR signal assignment	
Hydrogen	δ (ppm)	Multiplicity	Carbon	δ (ppm)
1 + NH^+^	1.47	Quadruplet	1	10.25–10.15
2	2.75–3.70	Multiplet	2	45.75–46.49
3	3.39	Triplet	3	58.27
4	1.51–2.01	—	4	23.81
5	1.28–1.86	—	5	24.64
6	1.44–1.91	—	6	24.46
7	1.68–1.86	—	7	29.91
8	4.90	Triplet	8	52.04
9	2.98	Singlet	9	33.24
10	—	—	10	171.46
11	—	—	11	132.37
12	7.72	Singlet	12	129.54
13	—	—	13	135.80
14	—	—	14	133.97
15	7.89	Doublet	15	127.79
16	7.45	Doublet	16	130.51

δ: chemical shift.

In order to increase the confidence on the NMR characterization, a HMBC experiment was performed in order to establish correlations over 2 heteronuclear bonds (SI.3).

The combination of UHPLC-HRMS and NMR experiments allow the complete identification of the compound. Nevertheless, an additional analytical technique was applied in order to unequivocally confirm the identity of U-49900.

#### Single-crystal X-ray diffraction

The unambiguous confirmation of the structure of U-49900 was performed by single-crystal X-ray diffraction analysis. Crystal evaluation and diffraction data were collected by using an Agilent Supernova diffractometer equipped with an Atlas CCD detector. No instrument or crystal instabilities were observed during data collection. Structure of U-49900 was solved by charge flipping methods using Superflip and refined by the full-matrix method on the basis of F^2^ with the program SHELXL-2013, using the OLEX software package^21–23^. Absorption correction based on the multiscan method was applied^24^. The graphic was performed with the Diamond visual crystal structure information system software^25^. Crystal data and structure refinement information are summarized in Table 2. All non-hydrogen atoms were refined anisotropically and the H atoms were positioned geometrically, assigned isotropic thermal parameters and allowed to ride on their respective parent carbon atoms.

**Table 2:** Crystallographic data for U-49900.

Parameter	Data
Empirical formula	C_18_H_26_Cl_2_N_2_O·HCl·H_2_O
Formula weight	411.78
Temperature (K)	200.0 (10)
Crystal system	Triclinic
Space group	P-1
Unit cell dimensions	
*a*, Å	7.30052 (13)
*b*, Å	8.14672 (13)
*c*, Å	18.8525 (3)
α, °	78.9257 (14)
β, °	82.3114 (14)
γ, °	66.9588 (16)
Volume (Å^3^)	1010.46 (3)
Z	2
ρ_calc_ (mg/mm^3^)	1.353
Absorption coefficient µ (mm^−1^)	4.218
F(000)	436.0
Crystal size (mm^3^)	0.174 × 0.12 × 0.095
Radiation	CuKα (λ = 1.54184)
2Θ range for data collection (°)	9.58 to 134.152
Index ranges	−8 ≤ *h* ≤ 8
	−9 ≤ *k* ≤ 9
	−22 ≤ *l* ≤ 22
Reflections collected	27700
Independent reflections	3616 [R_int_ = 0.0321, R_sigma_ = 0.0153]
Absorption correction	Multi-scan
Refinement method	Full-matrix least-squares on *F* ^*2*^
Data/restrains/parameters	3616/0/232
Goodness of fit on *F* ^*2*^	1.040
Final R indices [I > 2σ(I)]	R_1_ = 0.0288
	wR_2_ = 0.0754
R indices (all data)	R_1_ = 0.0320
	wR_2_ = 0.0784
Largest difference in peak/hole (e·A^−3^)	0.30/−0.23

**Figure 8:**
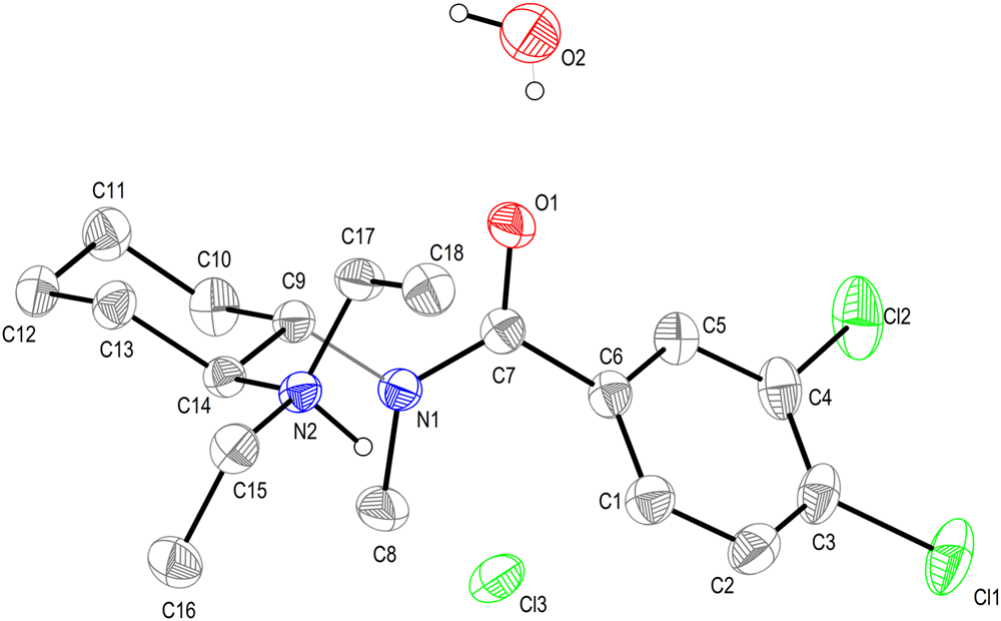
ORTEP representation (ellipsoids at the 50% probability level) of the U-49900 chloride protonated salt with the atom-numbering scheme. Selected bond lengths [Å]: N1-C7 1.356(9); N1-C8 1.466(9); N1-C9 1.479(9); N2-C14 1.528(9); N2-C15 1.512(9); C3-Cl1 1.732(9); C7-O1 1.234(9); C7-C6 1.502(11). Hydrogen atoms, except that of the protonated amine, have been omitted for clarity.

U-49900 structure was refined in the triclinic space group P-1 with cell dimensions of *a* = 73.0052(13) Å, *b* = 8.14672(13) Å, *c* = 18.8525(3) Å and α = 78.9257(14)°, β = 82.3114(14)° and γ = 66.9588(16)°. Figure 8 shows the ORTEP representation of the structure with the atom-numbering scheme. Protonation of the diethylamine group observed after performing NMR analyses was confirmed in the solid structure. This protonation produces the stereotopicity of the ethyl groups bonded to the nitrogen atom. However, the centrosymmetric P-1 space group evidences the racemic nature of the compound. Additionally, X-ray analysis demonstrated the high purity degree of this new substance and the high similarity to U-47700, and allowed the unequivocally identification of U-49900.

All data regarding single-crystal X-ray crystallography and structure refinement were included in the Cambridge Crystallographic Data Centre (CCDC). This information can be found free of charge in the CCDC 1546594 file available at www.ccdc.cam.ac.uk/data_request/cif.

#### Additional characterization

FTIR and UV analyses were performed in order to obtain additional information about the compound. These spectra can be found in Supplementary Information (SI.4 and SI.5, respectively). Moreover, the melting point range was determined and established to be between 169–171 °C. Finally, CD measurements were carried out to check the optical activity of U-49900. No Cotton effects were observed, confirming the racemic nature of this new drug.

### Psychoactive effects and availability of the drug as described by consumers

U-49900 is first mentioned online on November 2nd, 2016 on Bluelight, a popular drug forum, by a user announcing its availability and querying other users about possible effects. Users expressed concerns about its possible health hazards, as it is a close analogue to U-47700, which has been linked to damaged veins, damage to nasal and rectal passages, violent withdrawals, and other concerning effects^26, 27^. Most forum threads reference the same post made by the anonymous user^28^. In the post, the user reports concerning side effects like pain upon insufflation, loss of taste, loss of smell, neurologic pain on the left side of the body, loss of the sense of touch, and a foam-like discharge from the lungs.

Reports of effects are few and far between, as most users that inquire about effects are linked to the aforementioned post and subsequently refrain from consuming the substance^29^. A user on the Swedish forum Flashback reports classic opioid effects from a dose of 50 mg intravenous^30^. Users on the same Swedish forum have tried U-49900 at doses of 5–10 milligrams and report no activity, whereas U-47700 is active in those amounts.

## Discussion

In this work, the combination of different analytical techniques has allowed the unequivocal characterization of the novel opioid derivative U-49900. The use of HRMS, NMR and single-crystal X-ray diffraction has proved to be a powerful approach for the identification of NPS when reference standards are not available. Moreover, information obtained by GC-MS, FTIR and UV are also provided, in order to facilitate the identification of this compound in forensic laboratories with routine equipment. Although the present methodology could be used for the elucidation of most of organic compounds, it is especially useful for monitoring NPS due to the continuous on-going of these substances. This fact is the main handicap for obtaining reference standards, making the combination of powerful techniques necessary for unequivocal identification of NPS.

Although U-49900 is reported to be active, the doses taken are much larger than U-47700. Less than a year has passed since its first appearance on fora, but users’ experiences seem to indicate that U-49900 is yet another lackluster successor to U-47700^29^.

It is interesting to note that users of fora dedicated to NPS and other drug discussion seem to have a self-regulating system for novel compounds. Although these users are treading unknown territory, they are quick to share effects and side effects of NPS, and when such effects are concerning, they promptly share it with others and tend to steer clear of the substances discussed. One notable example is MT-45, a synthetic opioid developed in the 1970s by Dainippon Pharmaceutical Co^31^, which surfaced on internet shops late 2012^5, 32^, and has been involved in several non-fatal as well as fatal intoxications around the world. Users were quick to realize that among various side effects, MT-45 caused hearing loss and unconsciousness, and popularity dwindled well before its regulation in some European countries (like the UK and Czech Republic^33^) around 2015. It’s possible that this is due to the negative side effects being widely reported on and shared across NPS-focused fora. With U-49900 a similar trend appears to be taking place, as reports of use have been slow to surface, and most users that inquire about it are warned about potential side effects and resolve to stay away from the substance.

Possibly one of the most curious aspects of U-49900 is that, aside from bearing a striking structural similarity to U-47700, the name itself is a clear reference to this compound. It is, in fact, quite peculiar that the first appearance of this compound comes from a Chinese retailer^34^, and not from old studies or patents, as tends to be the case for most NPS. U-49900 is likely a deliberate attempt to find a similar compound to U-47700, and demonstrates at least a rudimentary grasp of pharmacology, seeing as the modification is minor, and in theory the compound should retain similar activity to its parent compound. A similar case is methoxetamine, an NPS structurally similar to ketamine and other arylcyclohexylamines, which surfaced on online shops before being mentioned on any scientific literature. There is an enigmatic interview with a British biochemist that went by the pseudonym “M”^35^ who claimed to have had a hand in the ideation and synthesis of this compound^36^. This suggests that for some NPS, the research and development stage is conducted in countries that have a market for NPS, and their production is then outsourced to Chinese laboratories. It is plausible that this is also the case for U-49900, and that there is a development process that took place in a European or North American country.

So far, there are no reported deaths related to U-49900, but at least 46 deaths were reported in the US alone with U-47700 being involved. Although this may appear to be a high figure, it pales in comparison to illicit fentanyl and its analogues, perhaps the closest contenders to the U-XXXXX family for use by opioid users, which have been involved in hundreds of deaths in the US alone^37–39^. It is clear that U-47700 is a harmful substance, with a much lower safety margin than other opioids or opiates, but the fact that the dosage is more manageable than fentanyl analogues, and the ratio of euphoric effects to respiratory depression suggests that users may be less prone to overdosing in the quest for the effects they are accustomed to with classical opiates.

This is especially important in US, where there is an important problem with opioid and opiate abuse. Users tend to start with pharmaceutical derivatives, such as codeine or tramadol, which are perceived as less harmful and their use is less stigmatized^40^. Some eventually migrate to more potent and euphoric derivatives, such as oxycodone or oxymorphone. When a dependence develops and the cost of the habit becomes prohibitive, some users may transition to illegal opioids and opiates, such as heroin, fentanyl analogues and/or novel opioid derivatives, such as non-pharmaceutical fentanyl, U-47700, or U-49900. These compounds tend to have lower safety margins than the more studied classical opiates. It is therefore important to evaluate the repercussions of scheduling novel opioids without contemplating future derivatives. It seems that scheduling these compounds as they surface has not been effective at curbing novel opioid abuse. A similar phenomenon was observed for synthetic cannabinoids, where the first generation (JWH-018, etc.) were prone to seizures, causing dependence, and other complications, but the latest generation, the result of several waves of prohibition, are more potent, more prone to causing complications, and have been involved in a striking amount of deaths^41–43^.

After the unequivocal identification of U-49900, metabolism and toxicological studies would be needed to get comprehensive information on this drug. The study of metabolic pathways of NPS is crucial for establishing the consumption biomarkers of these substances in urine for their monitoring in intoxication and overdoses cases^44^. However, obtaining urine or serum from intoxication cases is troublesome, as the source of the intoxication is commonly unknown. In the case of U-47700, a possible metabolism pathway has been proposed in literature^14^ (Supplementary Information SI.6), including 10 tentative metabolites. The major metabolites were the N-desmethyl and N,N-bisdesmetyl derivatives, which were found in a urine sample from a subject after U-47700 overdose.

In view of the difficulties of performing metabolism studies in humans, the *in vivo* approach using animals plays an important role. The use of different animal models (mice or rats) has demonstrated its efficiency for obtaining potential consumption markers of NPS in human urine samples^45, 46^. So, future research will be focused on metabolites identification of U-49900 using *in vivo* models (similarly to the analogue U-47700). Information provided on these or similar studies will be useful for monitoring this compound in consumers and in possible intoxication cases.

As a final note, a new opioid analogue related to U-47700 and identified as 3,4-dichloro-N-(2-(diethylamino)cyclohexyl)-N-methylbenzamide (also known as U-49900) has been identified and characterized in a drug sample from Spain. Characterization of the compound consisted of GC-MS analysis, UHPLC-HRMS, NMR, 2D-NMR analyses and single-crystal X-ray diffraction, unequivocally confirming its chemical structure. Additionally, FTIR, UV and CD spectra, as well as melting point range were measured in order to obtain a complete characterization of U-49900. The information reported in this work about this new opioid analogue will be useful to enhance the Early Warning Systems and for future toxicological studies.

It seems that demand for novel opioids does not decrease when a compound is scheduled, and if the supply for a certain substance dwindles, users are bound to seek an alternative. AH-7291 was dangerous, U-47700 has been proven to be more widespread and even more problematic, and the newest generation of derivatives, such as U-49900 and U-51754 do not seem to be of less concern. While it is clear that a solution needs to be found, perhaps quickly and systematically scheduling new compounds as they gain popularity can be complemented with other prevention measures in response to the growing problem of NPS abuse.

Future work on U-49900 will be focused on the metabolic pathway of this opioid in urine samples from *in vivo* experiments, as well as on the proposal of potential consumption markers. These studies will be useful for monitoring U-49900 in consumers and in suspicious intoxication cases.

## Methods

### Drug sample

The suspect sample (consisting on white powder) was submitted by an anonymous user to Energy Control for its analysis. Additional information about Energy Control can be seen elsewhere^47^.

#### Reagents and chemicals

For GC-MS analysis, HPLC-grade methanol (MeOH) was purchased from Panreac (Panreac, Barcelona, Spain). For UHPLC-HRMS analysis, HPLC-grade water was obtained by purifying demineralized water using a Milli-Q system from Millipore (Bedford, MA, USA). HPLC-grade methanol (MeOH), HPLC-grade acetonitrile (ACN), formic acid (HCOOH), acetone, and sodium hydroxide (NaOH) were acquired from Scharlau (Scharlab, Barcelona, Spain). Leucine enkephalin was purchased from Sigma-Aldrich (St. Louis, MO, USA). For NMR analysis, deuterated chloroform (CDCl_3_) and deuterated waters (D_2_O) were purchased from Sigma-Aldrich. For single-crystal X-ray analysis, GC ultra-trace analysis grade dichloromethane (stabilized with ethanol) and GC ultra-trace analysis grade n-hexane were purchased from Scharlau. For FTIR analysis potassium bromide (KBr) was purchased from Scharlau. For UV and CD analyses, ACN was purchased from Scharlau.

#### Sample treatment

For GC-MS analysis, 10 mg of bulk sample were sonication-assisted dissolved with 10 mL of MeOH during 15 min. The extract was then centrifuged to remove insoluble material and afterwards directly injected into GC-MS system.

For UHPLC-HRMS analysis, 10 mg of sample were extracted with 1 mL of acetone in an ultrasonic bath for 15 min, following the sample treatment described in literature^48, 49^. After centrifugation, the supernatant was ten thousand-fold diluted with HPLC-grade water, and 20 µL of the extract were injected in the UHPLC-HRMS system.

For NMR analysis, approximately 15 mg of sample were dissolved in 0.6 mL of CDCl_3_. Additionally, 15 mg of sample were dissolved in 0.6 mL of D_2_O in order to obtain a ^1^H-NMR spectrum without the protonated amine signal.

The recrystallization was performed by dissolving 10 mg of sample in 1 mL of dichloromethane in a glass vial, and dropwise addition of n-hexane, taking care to avoid mixing both solvents. The process was performed over-night, by diffusion of n-hexane in dichloromethane and thus, precipitating the compound and crystallizing it. Needle-shaped crystals were obtained and used for X-ray diffraction.

For FTIR analysis, the sample was mixed with KBr in a ratio 5:95 and homogenized in an agate mortar. Finally, the mixture was compressed under a pressure of 5000 kg/cm^2^.

For UV and CD analyses, the compound was dissolved in ACN at 10^−4^ M, preparing the appropriate volume of dissolution.

#### Instrumentation

For GC-MS analysis, an Agilent 7890B gas chromatograph was coupled to a 5977 A quadrupole mass spectrometer detector (Agilent Technologies, Santa Clara, CA, USA) using an electron ionization (EI) interface. The gas chromatograph was fitted with a G4513A auto-sampler injector (Agilent). Insert liners packed with silanized glass wool were used, and the injector and the interface were operated at 280 °C. 1 µL of sample was injected in split mode, with a split ratio 1:10, into a 30 m 0.25 mm i.d., 0.25 μm film thickness 5% phenylmethylsilicone column (HP-5MS, Agilent). Helium was used as carrier gas at a flow rate of 1 mL/min. The oven temperature was initially maintained at 90 °C for 2 min and programmed to reach 320 °C at 20 °C per min. It was finally maintained at 320 °C for 9.5 min (total run time was 21.5 min). The mass spectrometer was operated in electronic ionization mode at 70 eV. MS system worked in SCAN acquisition mode, acquiring from *m/z* 40 to 400 Da. Analytical data were acquired and processed using MassHunter B.06.00 (Agilent) operation software.

UHPLC-HRMS analysis was performed using an ACQUITY UPLC ultra-high performance liquid chromatography (UHPLC) system (Waters, Mildford, MA, USA) coupled to a XEVO G2 QTOF hybrid quadrupole time-of-flight (QTOF) mass spectrometer (Waters Micromass, Manchester, UK) with an orthogonal Z-spray electrospray ionization (ESI) interface operating in positive ionization mode. The chromatographic separation was performed using a CORTECS C18 (Waters) 2.7 µm particle size analytical column 100 × 2.1 mm at a flow rate of 0.3 mL/min. The column temperature was set to 40 °C. The mobile phases used were H_2_O with 0.01% HCOOH (A) and MeOH with 0.01% HCOOH (B). The mobile phase gradient was performed as follows: 10% of B at 0 min, 90% of B at 14 min linearly increased, 90% of B at 16 min, and finally 10% B at 18 min in order to return to initial conditions. The injection volume was 10 µL. Nitrogen (Praxair, Valencia, Spain) was used as desolvation and nebulizing gas. The desolvation gas flow was set at 1000 L/h. The TOF resolution was ~20000 at FWHM at *m/z* 556. The range acquired by the MS system was from *m/z* 50 to 1000. A capillary voltage of 0.7 kV and a cone voltage of 20 V were used during all the chromatographic run. Argon 99.995% (Praxair, Valencia, Spain) was used as a collision gas. The interface temperature was set to 650 °C and the source temperature to 120 °C. For MS^E^ experiments, two acquisition functions with different collision energy were created. The low energy function (LE) used a collision energy of 4 eV in order to obtain information about the protonated molecule and adducts (if present), while the high energy function (HE) applied a collision energy ramp from 15 to 40 eV, in order to promote fragmentation of the compounds^48^. Calibration of the mass-axis was performed daily from *m/z* 50 to 1000 using a 1:1 mixture of 0.05 M NaOH:5% HCOOH, diluted 1:25 with ACN:H_2_O 80:20 mixture. For accurate mass measurement, a 2 µg/mL leucine enkephalin solution in ACN:H_2_O 50:50 with 0.1% HCOOH was used as lock-mass, pumped at a flow rate of 20 µL/min. The leucine enkephalin protonated molecule (*m/z* 556.2771) was used for recalibrating the mass axis and ensure an accurate mass during all the chromatographic run. MS data were acquired in centroid mode using MassLynx data station operation software, version 4.1 (Waters).

NMR analyses were performed using a Bruker Ascend 400 MHz spectrometer equipped with a SampleCase autosampler (Bruker, Etlingen, Germany), performing data acquisition at 303 K using CDCl_3_. The residual solvents signals at δ = 7.24 ppm for ^1^H (CHCl_3_) and at δ = 77.23 ppm for ^13^C (CDCl_3_) were used as internal references. Characterization of the compound was performed using 5 gradient-enhanced experiments: ^1^H-NMR, ^13^C-NMR, correlated spectroscopy (COSY), heteronuclear single quantum coherence (HSQC), and heteronuclear multiple bond correlation (HMBC). NMR experiment data were collected using the Bruker Icon NMR 5.0.5 software (Bruker). MestreNova program was used for raw data processing (Mestrelab Research, Santiago de Compostela, Spain).

For single-crystal X-ray diffraction crystallography, an Agilent SuperNova diffractometer (Agilent Technologies) was used. The diffractometer was equipped with an Atlas CCD detector (Agilent Technologies), and CuKα radiation (λ = 1.54184 Å) was used. Sample was kept at 199.95 K during data collection. Experimental data was acquired using the SHELXS-2013 software (Yale University, New Haven, CT, USA), using the OLEX software package (Olex AS, Trondheim, Norway).

For FTIR analysis, a Jasco FT/IR-6200 FTIR spectrometer (Jasco Inc., Easton, MD, USA) was used. Data acquisition was performed at 23 °C between 4000 and 400 cm^−1^, with a resolution of 4 cm^−1^ and performing 32 acquisitions.

For UV and CD analyses, a Jasco J-810 spectrophotometer (Jasco Inc., Easton, MD, USA) was used. Data acquisition was performed at 23 °C between 200 and 400 nm, with a resolution of 1 nm, a scanning speed of 100 nm/min and performing 2 acquisitions.

## Electronic supplementary material

Supplementary Information
