# Supplementary material for: Updating the list of known opioids through identification and characterization of the new opioid derivative 3,4-dichloro-N-(2-(diethylamino)cyclohexyl)-N-methylbenzamide (U-49900)
Source: Sci Rep. 2017 Jul 24;7:6338. doi: 10.1038/s41598-017-06778-9 (PMC5524693; doi:10.1038/s41598-017-06778-9)
Supplement: Supplementary file 1 — Supplementary Information [file 41598_2017_6778_MOESM1_ESM.pdf]

Supplementary Information

Updating the list of known opioids through identification and characterization of the new opioid derivative 3,4-dichloro-N-(2-(diethylamino)cyclohexyl)-N-methylbenzamide (U-49900).

D. Fabregat-Safont[1], X. Carbón[2], M. Ventura[2], I. Fornís[2], E. Guillamón[3], JV. Sancho[1], F. Hernández[1], M. Ibáñez[1]

[1] Research Institute for Pesticides and Water, University Jaume I, Avda. Sos Baynat s/n, 12071, Castellón, Spain. [2] Energy Control (Asociación Bienestar y Desarrollo), c/ Independencia 384, 08041, Barcelona, Spain. [3] Departament de Química Física I Analítica, University Jaume I, Avda. Sos Baynat s/n, 12071, Castellón, Spain. Correspondence and request for materials should be addressed to M.I. (email: ibanezm@uji.es).

- **SI.1.** MS/MS spectra
- **SI.2.** ^{1}H-NMR spectra acquired using $CDCl_3$ and D_2O comparison.
- **SI.3.** HMBC spectrum
- **SI.4.** FTIR spectrum
- **SI.5.** UV spectrum
- **SI.6.** U-47700 metabolic pathway

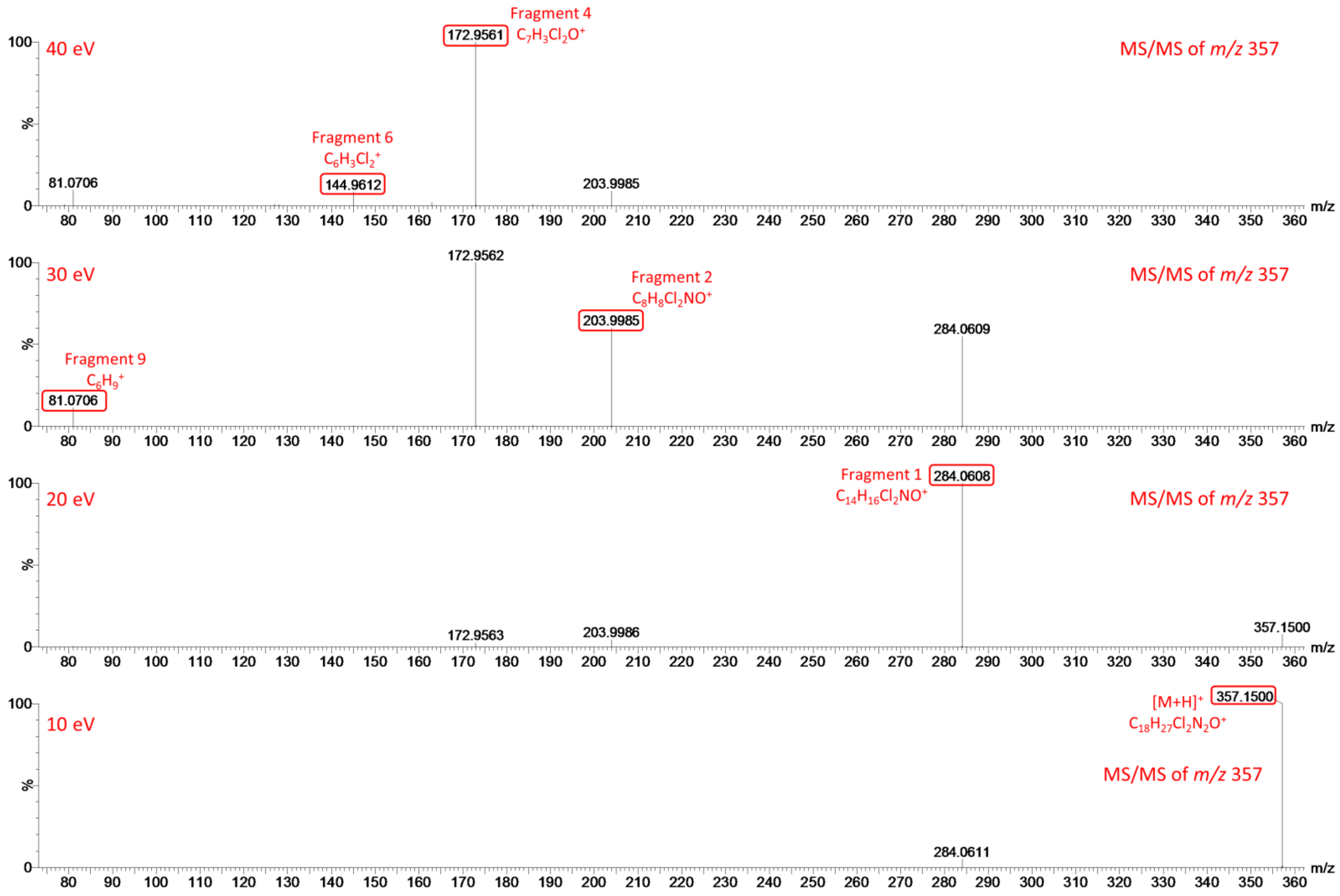

SI.1. MS/MS spectra of U-49900 at 10, 20, 30 and 40 eV collision energy.

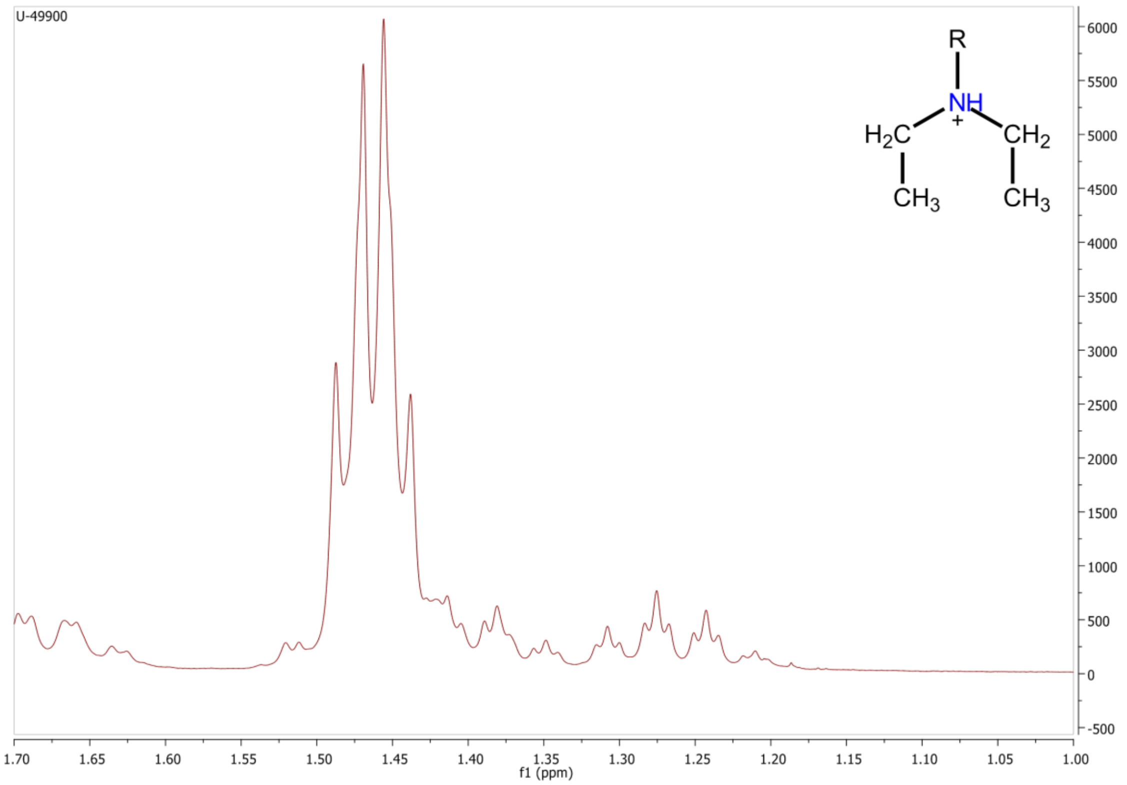

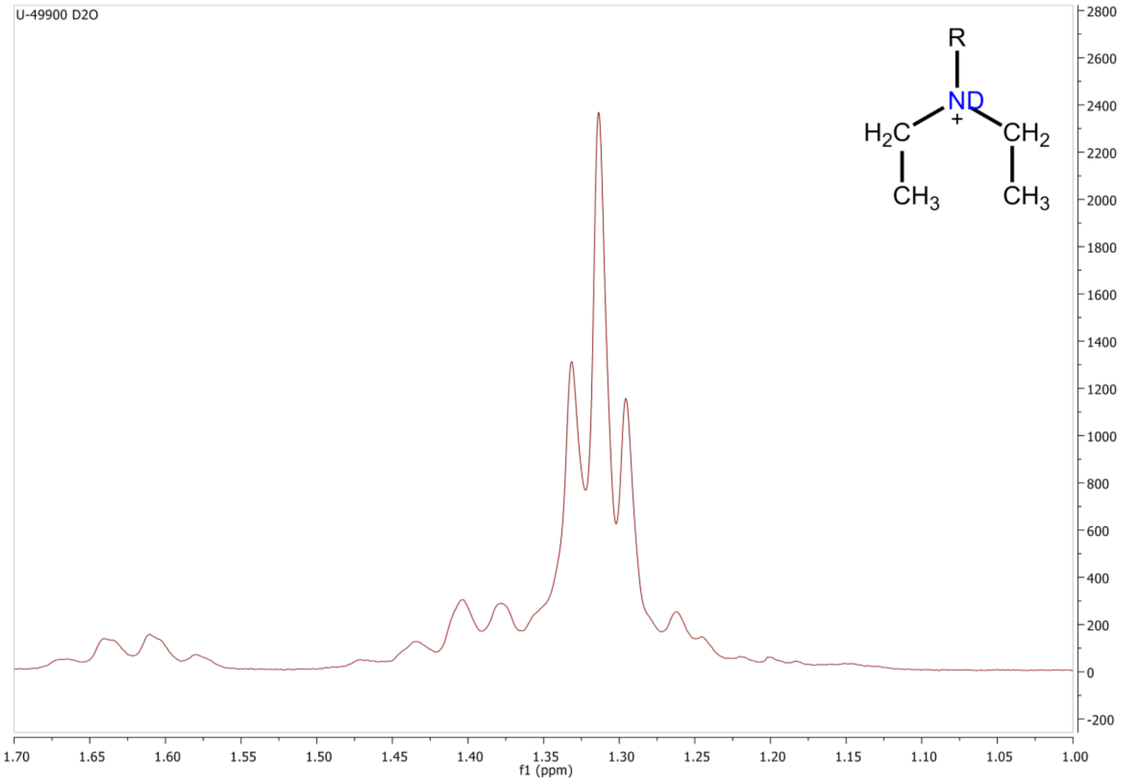

SI.2. ^{1}H-NMR signal for hydrogens of the methyl groups of diethylamine group. **Top:** Signal obtained when using $CDCl_3$ for dissolve the sample. **Bottom:** Signal obtained when using D_2O for dissolving the sample.

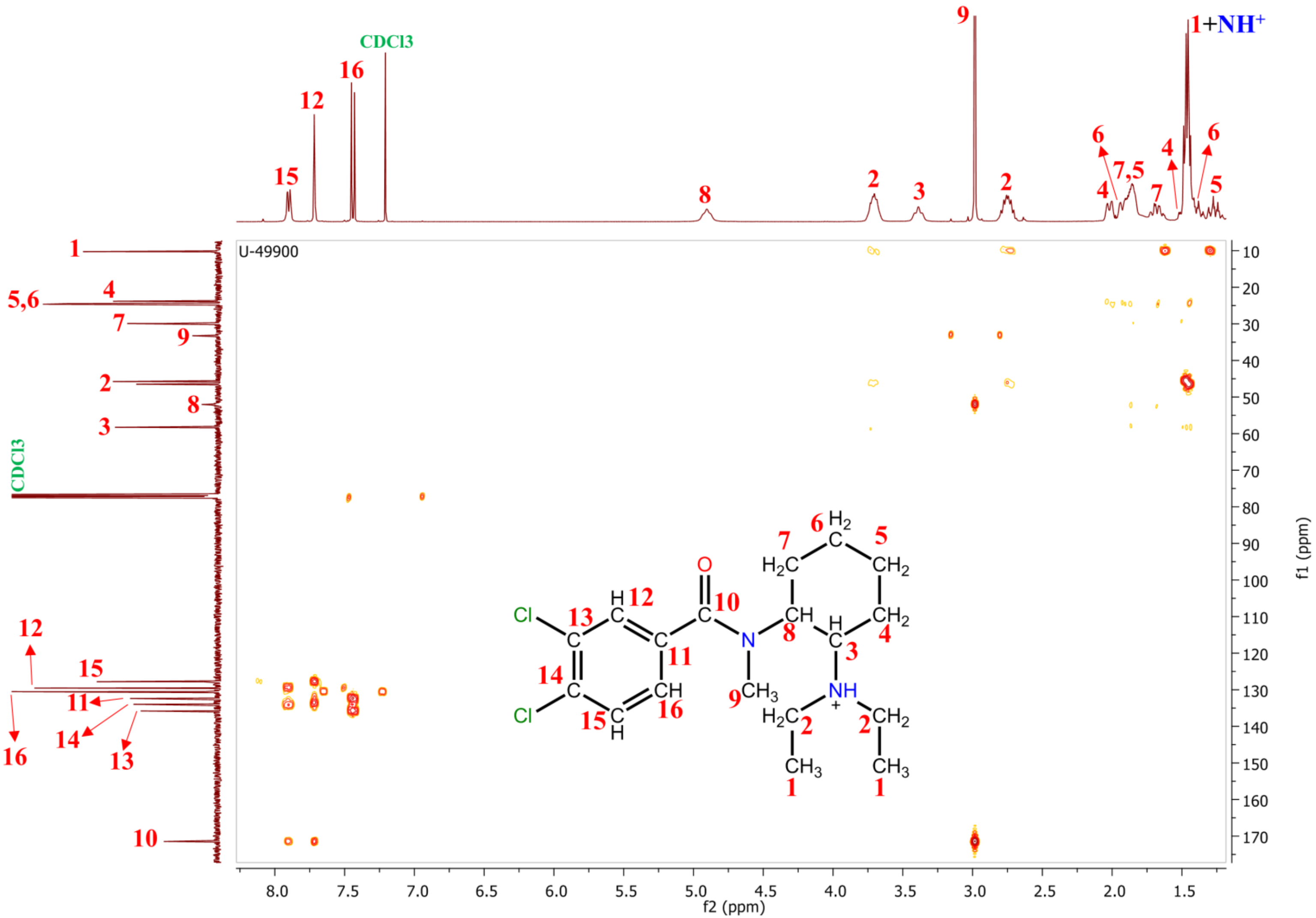

SI.3. HMBC spectra of U-49900. Correlations over 2 bonds are showed between ^{1}H and ^{13}C-NMR signals.

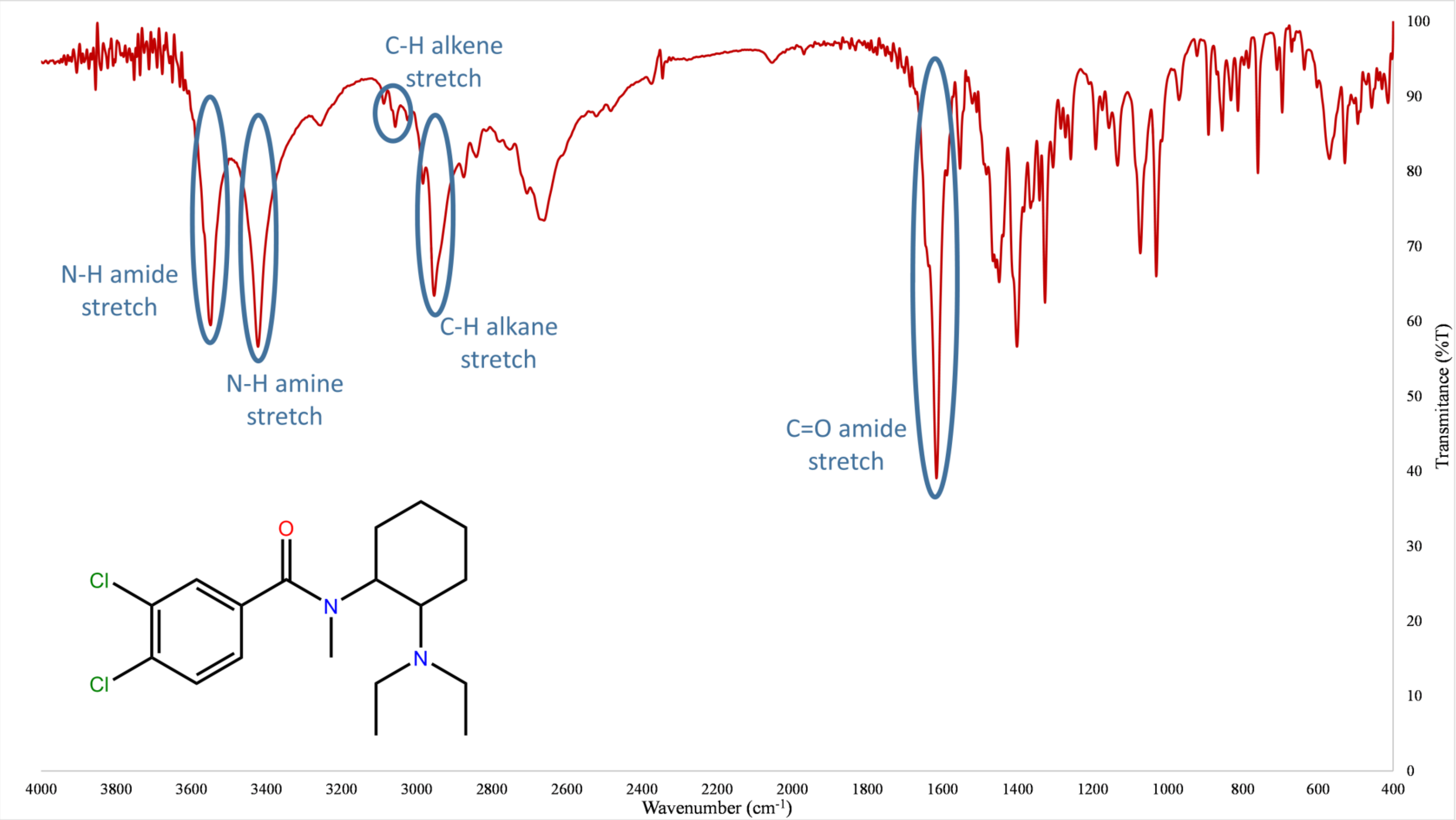

SI.4. FTIR spectrum of U-49900. C=O band of the amide group is observed at 1600-1650 cm^{-1}. Aliphatic C-H bands are observed down to 3000 cm^{-1}. Aromatic C-H bands are observed up to 3000 cm^{-1}. N-H band of the amine is observed around 3400 cm^{-1}. N-H band of the amide is observed around 3500 cm^{-1}.

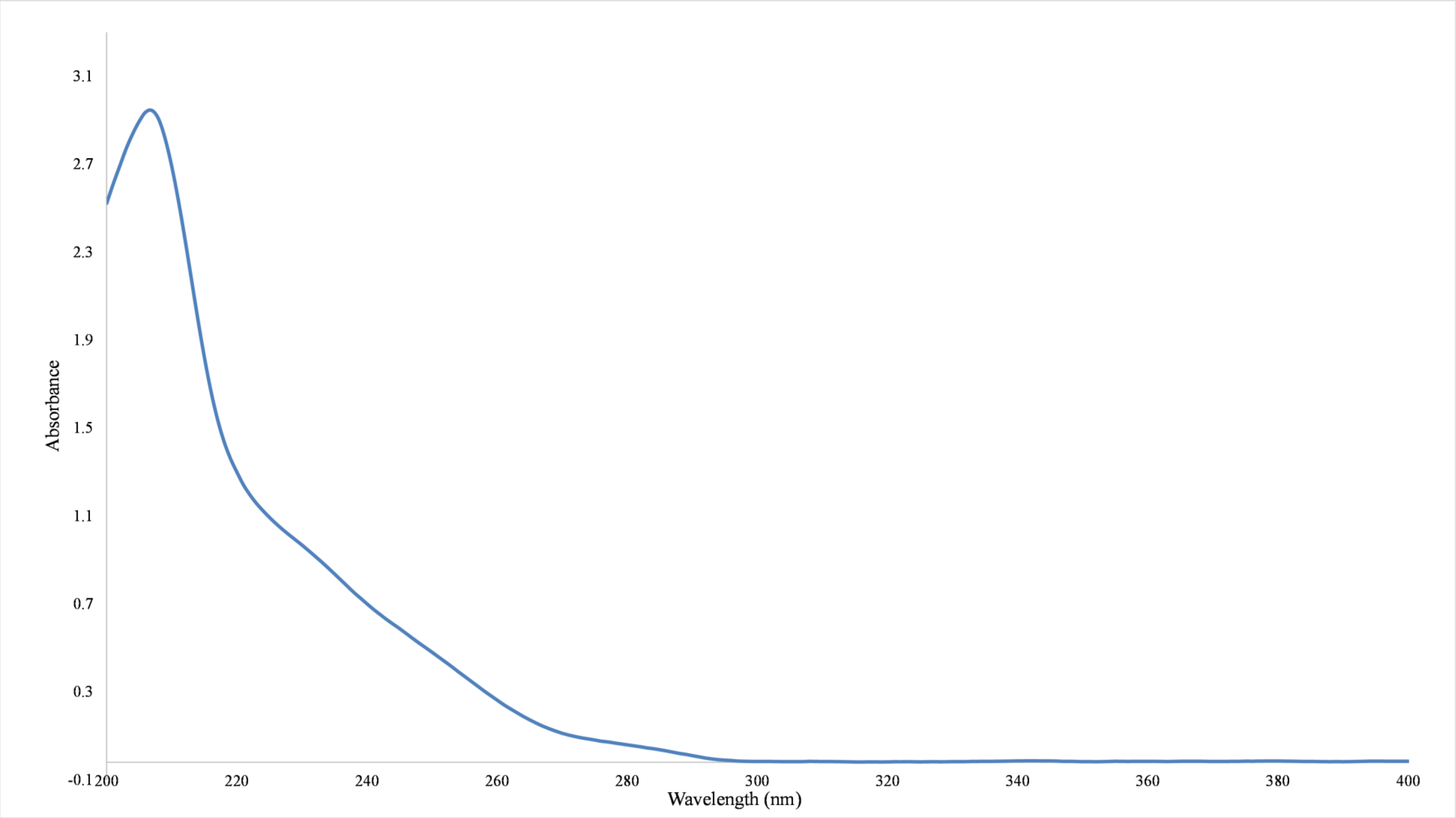

SI.5. UV spectrum of the U-49900

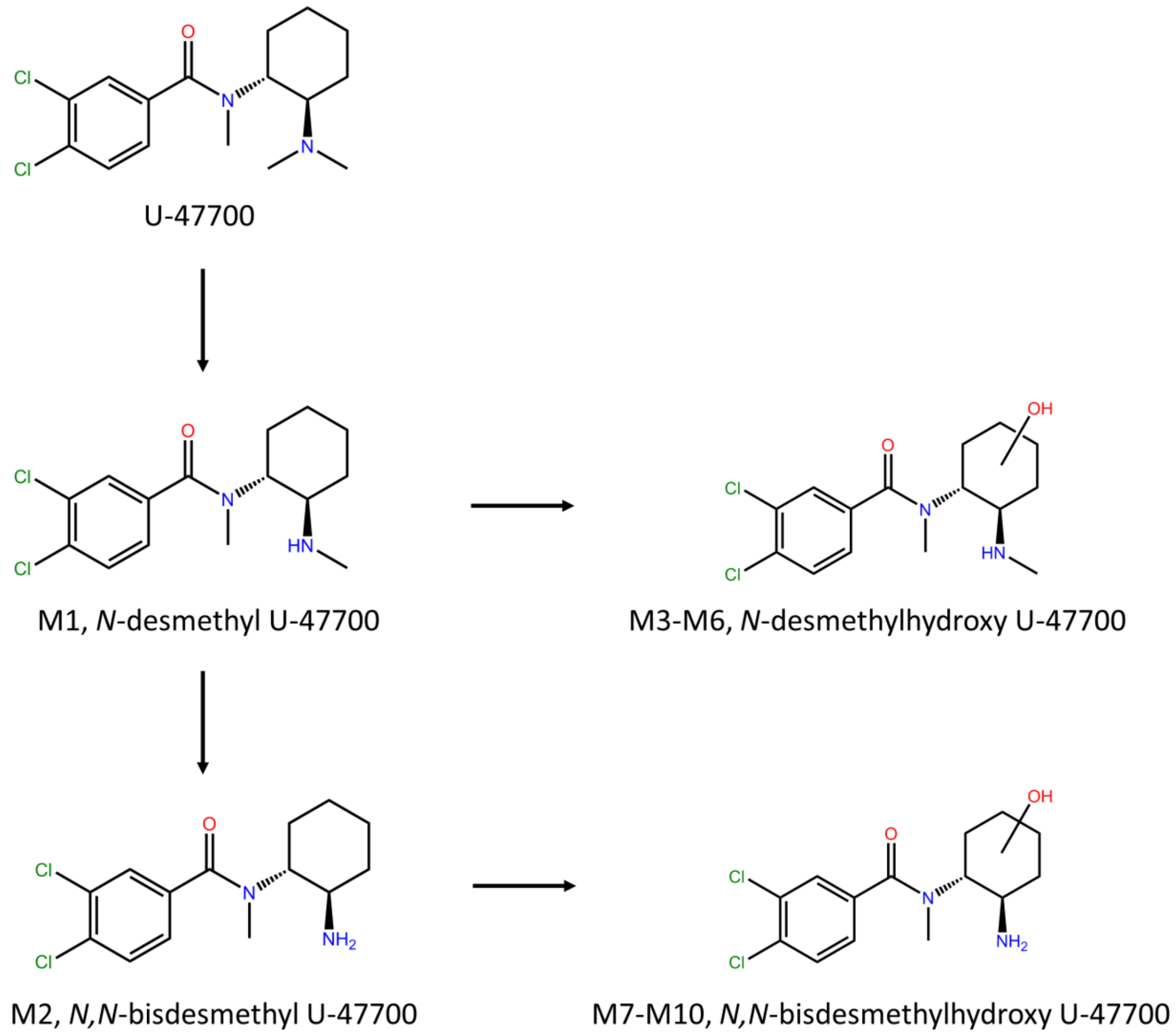

SI.6. Proposed metabolic pathway of U-47700.
